# Editorial: Data-driven clinical biosignatures and treatment for neurodegenerative diseases, volume II

**DOI:** 10.3389/fnins.2024.1396702

**Published:** 2024-03-18

**Authors:** Nizhuan Wang, Lei Chen, Wei Kong, Chung Y. Hsu, I-Shiang Tzeng

**Affiliations:** ^1^Department of Chinese and Bilingual Studies, The Hong Kong Polytechnic University, Kowloon, Hong Kong SAR, China; ^2^College of Information Engineering, Shanghai Maritime University, Shanghai, China; ^3^College of Medicine, Graduate Institute of Clinical Medical Science, China Medical University, Taichung, Taiwan; ^4^Department of Statistics, School of Business, National Taipei University, New Taipei, Taiwan

**Keywords:** speech analysis, glymphatic system, early diagnosis, digital therapy, sex difference, neurodegenerative disease

The pursuit of reliable, effective, and convenient biosignatures is paramount for the early diagnosis of neurodegenerative diseases (NDD), offering crucial insights into optimal treatment timing and disease progression (Wang et al., 2023). Recent advancements have led to the discovery of novel biosignatures and treatment modalities for NDD. For instance, Zetterberg's team identified a plasma p-tau217 immunoassay that accurately detects Alzheimer's Disease (AD), comparable to cerebrospinal fluid (CSF) biomarkers (Ashton et al., 2024). Similarly, Hansson et al. demonstrated the clinical efficacy of blood plasma p-tau217 for AD pathology detection, surpassing FDA-approved CSF tests (Barthélemy et al., 2024). Additionally, studies underscore the role of metabolic waste accumulation, particularly in AD, with neurons regulating brain clearance through the glymphatic system (Jiang-Xie et al., 2024). In NDD treatment, Tsai's team found that multisensory gamma stimulation enhances CSF dynamics in AD mouse models, while vasoactive intestinal peptide interneurons facilitate glymphatic clearance (Murdock et al., 2024). This Research Topic comprises five following papers, categorized into Speech Analysis for Early Diagnosis of NDD, Neurobiological Markers in NDD, and Digital Therapy Progress of NDD.

## Speech analysis for early diagnosis of NDD

Innovative speech analysis techniques hold significant promise for the early diagnosis of NDD. For instance, García-Gutiérrez et al. employ spontaneous speech analysis combined with artificial intelligence (AI) to identify individuals in the preclinical stages of mild cognitive impairment (MCI), a precursor to AD. By analyzing voice recordings of MCI patients, the study successfully correlates acoustic features with amyloid status in CSF, a biomarker for AD. Their AI model achieved 75% accuracy and an AUC of 0.79 in predicting amyloid status, surpassing conventional neuropsychological tests. This underscores the potential of automated voice analysis to offer valuable insights into AD biomarkers during early stages, facilitating the identification of high-risk subjects.

In parallel, Xu et al. introduce enhancements to speaker diarization, a crucial preprocessing step for diagnosing cognitive impairments using speech-based assessments. These enhancements address challenges arising from acoustic mismatches caused by far-field microphones. Through the integration of multi-scale channel interdependence speaker embedding and pairwise similarity measures, the proposed method outperforms conventional systems in diarization accuracy, even across language-, age-, and microphone-mismatch scenarios. Notably, the method enables the hypothesis of speaker-turn timestamps, enhancing adaptability to datasets lacking timestamp information.

## Neurobiological markers in NDD

The presented Research Topic comprises two studies elucidating neurobiological markers and sex differences in NDD, focusing on AD and Parkinson's disease (PD), respectively. For instance, Zhong et al. investigate the role of the glymphatic system in AD, revealing impairment in AD patients. Utilizing diffusion tensor imaging analysis, they assess glymphatic system activity across different AD stages in 300 subjects, finding significant differences correlating with cognitive decline and clinical scales, particularly in the left hemisphere. The study proposes the glymphatic system activity index (ALPS-index) and fractional anisotropy values as potential biomarkers for AD progression, aiding in treatment targets and clinical diagnosis.

Meanwhile, Cai et al. explore sex differences in myelin content in PD and its clinical implications. Using myelin water fraction imaging in 33 PD subjects, they identify sex-specific myelin variations in various white matter regions, associated with differences in motor symptomatology. Tremor and bradykinesia are more prevalent in females, while rigidity and axial symptoms are more common in males. These findings underscore the importance of further investigating the role of biological sex in myelin pathology and clinical presentation in PD.

## Digital therapy progress of NDD

Addressing the growing challenge of AD in the context of global population aging, Zhang et al. investigate the effectiveness of digital therapy as a novel approach for treatment and monitoring. Recognizing the limitations of traditional modalities, the study conducts a comprehensive review, focusing on AD and geriatric cognition. Evaluation of digital therapy, particularly employing functional near-infrared spectroscopy and electroencephalography monitoring, highlights biomarkers such as theta coherence, alpha and beta rhythms, and oxyhemoglobin, crucial for monitoring cognitive status. The review underscores the favorable efficacy of digital treatment based on biomarker monitoring, as evidenced by numerical changes pre- and post-treatment.

In summary, the studies in this Research Topic advance early diagnosis, elucidate neurobiological mechanisms, and emphasize the significance of sex-specific considerations in disease pathology and clinical therapy, with regard to the NDD. These insights hold potential for informing personalized diagnostic and therapeutic approaches of NDD in the near future.

## Author contributions

NW: Writing—original draft, Writing—review & editing. LC: Writing—review & editing. WK: Writing—review & editing. CH: Writing—review & editing. I-ST: Writing—review & editing.
